# Replication of Previous Findings? Comparing Gray Matter Volumes in Transgender Individuals with Gender Incongruence and Cisgender Individuals

**DOI:** 10.3390/jcm10071454

**Published:** 2021-04-01

**Authors:** Benjamin Clemens, Mikhail Votinov, Andrei Alexandru Puiu, Andre Schüppen, Philippa Hüpen, Josef Neulen, Birgit Derntl, Ute Habel

**Affiliations:** 1 Department of Psychiatry, Psychotherapy and Psychosomatics, Faculty of Medicine, RWTH Aachen University, 52062 Aachen, Germany; mvotinov@ukaachen.de (M.V.); apuiu@ukaachen.de (A.A.P.); rhuepen@ukaachen.de (P.H.); uhabel@ukaachen.de (U.H.); 2 Institute of Neuroscience and Medicine 10, Research Centre Jülich, 52428 Jülich, Germany; 3 Interdisciplinary Center for Clinical Research (IZKF), Faculty of Medicine, RWTH Aachen University, 52062 Aachen, Germany; aschueppen@izkf.rwth-aachen.de; 4 Division for Clinical and Cognitive Sciences, Department of Neurology, Faculty of Medicine, RWTH Aachen University, 52062 Aachen, Germany; 5 Department of Gynecological Endocrinology and Reproductive Medicine, Faculty of Medicine, RWTH Aachen University, 52062 Aachen, Germany; jneulen@ukaachen.de; 6 Department of Psychiatry and Psychotherapy, University of Tübingen, 72074 Tübingen, Germany; birgit.derntl@med.uni-tuebingen.de; 7 LEAD Graduate School and Research Network, University of Tübingen, 72072 Tübingen, Germany

**Keywords:** gender incongruence (GI), gray-matter volumes (GMV), magnetic resonance imaging (MRI), neuroanatomy, transgender

## Abstract

The brain structural changes related to gender incongruence (GI) are still poorly understood. Previous studies comparing gray matter volumes (GMV) between cisgender and transgender individuals with GI revealed conflicting results. Leveraging a comprehensive sample of transmen (*n* = 33), transwomen (*n* = 33), cismen (*n* = 24), and ciswomen (*n* = 25), we employ a region-of-interest (ROI) approach to examine the most frequently reported brain regions showing GMV differences between trans- and cisgender individuals. The primary aim is to replicate previous findings and identify anatomical regions which differ between transgender individuals with GI and cisgender individuals. On the basis of a comprehensive literature search, we selected a set of ROIs (thalamus, putamen, cerebellum, angular gyrus, precentral gyrus) for which differences between cis- and transgender groups have been previously observed. The putamen was the only region showing significant GMV differences between cis- and transgender, across previous studies and the present study. We observed increased GMV in the putamen for transwomen compared to both transmen and ciswomen and for all transgender participants compared to all cisgender participants. Such a pattern of neuroanatomical differences corroborates the large majority of previous studies. This potential replication of previous findings and the known involvement of the putamen in cognitive processes related to body representations and the creation of the own body image indicate the relevance of this region for GI and its potential as a structural biomarker for GI.

## 1. Introduction

Gender identity development is a complex process involving multifactorial interactions among genetic, hormonal, social, and psychological factors. As a result of this complex process, there are different gender identities, including, among others, female, male, nonbinary, agender, gender nonconforming, gender fluid, intersex, pangender, genderqueer, or androgynous. Whereas biological sex (i.e., the sex assigned to an individual at birth on the basis of the anatomy of the reproductive system) and gender identity (i.e., the subjective identification of an individual as male, female, or one of the other gender identities) coincide in most people, there are individuals who do not identify with their biological sex. The phenomenon of gender incongruence (GI) is more widespread and relevant than often thought. Individuals for whom gender identity is different from their biological sex are referred to as transgender, whereas the term cisgender describes individuals whose biological sex and gender identity coincide. Recent media attention to transgender and GI has increased substantially, with changes in laws and attempts to reduce societal discrimination allowing more people to openly identify as gender incongruent and seek treatments such as gender-affirming hormone therapy (GAHT) and gender-affirming surgery. A large portion of transgender individuals are constantly exposed to high levels of social stress and suffer from severe discomfort [1,2,3,4]. Patients affected by GI have to deal with difficulties in accessing relevant healthcare services and adequately trained primary care providers. In an attempt to reduce stigmatization and facilitate access to relevant healthcare needs, the 11th version of the International Classification of Diseases (ICD-11) added “gender incongruence (GI)” as a diagnosis to the sexual health section and removed “gender dysphoria” from the mental health section [5]. With respect to the prevalence of being transgender in the general population, two recent epidemiological studies conducted in the Netherlands and Belgium revealed that 1.1% of individuals with male birth-assigned sex and 0.8% of individuals with female birth-assigned sex reported an incongruent gender identity [6,7]. Evaluating worldwide prevalence rates in a meta-analytic approach, Arcelus et al. reported a rate of around 4.6 per 100,000 for being transgender [8].

The neurobiological mechanisms underlying GI and more specifically changes in brain structure and function are still poorly understood. A neurobiological parameter that might be of crucial importance is the study of brain anatomical differences. Several magnetic resonance imaging (MRI) studies comparing transgender individuals with GI and cisgender individuals found brain structural differences in the putamen, the thalamus, and the angular and the insular gyri [9,10,11]. Furthermore, other studies showed a strong overlap of brain structures in individuals sharing the same gender identity [12,13,14,15]. Whereas some previous studies in transgender individuals revealed structural brain patterns more consistent with the gender identity in hypothalamus [12,14], left pre- and postcentral gyri [15], and the nucleus accumbens [16], others demonstrated structural brain patterns more consistent with the biological sex in putamen, precentral gyrus [10], and the frontal cortex [17]. Overall, findings on brain structural differences between cis- and transgender individuals are very heterogeneous and, in many cases, difficult to interpret due to potential confounders of sexual orientation [18,19], genetic factors [2], hormonal factors [20], and small sample sizes [8]. Some researchers believe that differences in cortical development and in β-estrogen receptor efficiency might primarily influence the development of unique brain structural phenotypes for specific gender identity subtypes during early neurodevelopmental phases [12,14,21]. Another approach postulates that the human brain is best described as a mosaic of female and male characteristics, refuting the oversimplified concept of a dichotomous “female” or “male” brain [22,23]. Despite such interesting findings and theoretical efforts, the precise brain anatomical correlates that most reliably and specifically indicate GI and, thus, differentiate between transgender and cisgender individuals remain elusive.

The majority of previous studies focused on interpreting structural differences in the context of sexual differentiation, primarily trying to clarify whether the brain of transgender participants resembles that of their biological sex or that of their experienced gender identity. The present study aims to replicate previous findings and identify anatomical regions which differ between transgender individuals with GI and cisgender individuals. We identified a set of candidate regions from previous studies and examined whether we can replicate these findings in our own sample of cis- and transgender participants. Our hypothesis is that we can identify one or several distinct anatomical regions for which gray matter volume (GMV) differences from previous studies can be replicated in the present study. Using a sample of cismen, ciswomen, transmen, and transwomen, we focus solely on differences in GMV, since this parameter has been examined most extensively, with conflicting results for several anatomical structures. Successful replication of previous findings for a specific anatomical region would help in clarifying the aforementioned contradictory results. An overarching aim of this study is to facilitate the identification of candidate brain regions as structural biomarkers in GI.

## 2. Experimental Section

### 2.1. Participants

The MRI data presented here were recorded in the context of a larger research project comprising several functional MR tasks and resting-state fMRI, which are reported elsewhere [22,24,25,26]. Transgender participants were either transmen (TM), describing individuals born with the biological sex of a woman but identifying as men, or transwomen (TW), describing individuals born with the biological sex of a man but identifying as women. Cisgender participants are referred to as CW for cisgender women and CM for cisgender men. Since we aimed to assess specific gender identity effects irrespective of the biological sex, we combined CW and CM to form the cis group and TM and TW to form the trans group. For direct comparability with previous studies and to enable a more in-depth analysis, GMV is also compared among the four groups separately. In total, 115 participants took part in the present study, including 24 CM, 25 CW, 33 TM, and 33 TW (*n* = 66 trans; *n* = 49 cis). All cisgender participants were recruited via public announcement around Aachen (Germany). TM and TW were recruited in self-help groups and at the Department of Gynecological Endocrinology and Reproductive Medicine of the RWTH Aachen University Hospital, Germany. All cisgender participants reported heterosexual orientation, whereas sexual orientation was not systematically assessed in transgender participants. This was partly because the majority of transgender participants were unable or unwilling to describe themselves as either hetero- or homosexual. Transgender participants either started already with or firmly declared their intention of undergoing GAHT in the future, stated a strong sense of belonging to the opposite sex, and also expressed the desired gender identity in everyday life. All transgender participants fulfilled diagnostic criteria for GI, as diagnosed by a board-certified mental-health professional at the Department of Psychiatry, Psychotherapy, and Psychosomatics of the RWTH Aachen University Hospital, Germany. The German version of the Structured Clinical Interview of the fourth edition of the Diagnostic and Statistical Manual of Mental Disorders (DSM-IV) [27] was used to ensure the exclusion of participants with mental health diagnoses unrelated to GI. For all cis- and transgender participants, further exclusion criteria were presence of neurological disorders, other medical conditions affecting the brain metabolism, and first-degree relatives with a history of mental disorders. The local Ethics Committee of the Medical Faculty of RWTH Aachen University approved the study (EK 088/09). Participants were financially reimbursed and gave their written informed consent for participation.

### 2.2. Data Acquisition

The MRI data were acquired using a 3 T Siemens Trio MR Scanner (Siemens Medical Systems, Erlangen, Germany) at the Department of Psychiatry, Psychotherapy, and Psychosomatics of the RWTH Aachen University Hospital. Whole-brain images were obtained from each participant using the following parameters: T1-weighted Magnetization Prepared-Rapid Gradient Echo (MP-RAGE) 3D measurement; time repetition (TR) = 1900, time echo (TE) = 2.52, time inversion (TI) = 900; fli angle (α) = 9°, Field of View (FoV) = 250 mm^2^, voxel size: 1 × 1 × 1 mm^3^, slices = 176.

### 2.3. Data Preprocessing

Imaging data were preprocessed using the SPM12 software (Wellcome Department of Imaging Neuroscience Group, London, UK; http://www.fil.ion.ucl.ac.uk/spm (accessed on 15 September 2020)) running under MATLAB 2017b (The MathWorks, Natick, MA, USA) and the CAT12 toolbox, version 12.7 [28]. All anatomical images were manually reoriented to the intercommissural plane to improve spatial registration of anatomical images. Furthermore, images were corrected for field intensity inhomogeneities and spatially normalized into standard space. The images were segmented into gray matter (GM), white matter (WM), and cerebrospinal fluid (CSF), and the segmented tissue was modulated with Jacobian determinants. The modulated gray-matter volumes were smoothed with a Gaussian kernel of 8 mm full width at half maximum (FWHM), which represents a good kernel for detecting morphometric differences in both small and larger neural structures [29,30]. Lastly, the normalized, modulated, and smoothed gray-matter segments of all 115 participants were included for further statistical analyses.

### 2.4. ROI Selection and Definition

We searched the existing literature as of December 2020 with the goal of identifying the most frequently reported ROIs, i.e., regions that were repeatedly found in previous studies, albeit with contrasting results for comparisons between cisgender and transgender individuals. The following search terms were used: brain AND (transgender OR transsexual OR gender dysphoria OR gender incongruence) AND (magnetic resonance imaging OR MRI OR voxel-based morphometry OR VBM). Studies were selected for further inspection if they employed either whole-brain or ROI analyses and if they included GMV as the primary outcome parameter. Studies examining children and studies only comparing total brain volumes or cortical thickness were excluded. This search process yielded 11 studies [9,10,11,15,17,31,32,33,34,35,36].

**Figure 1:**
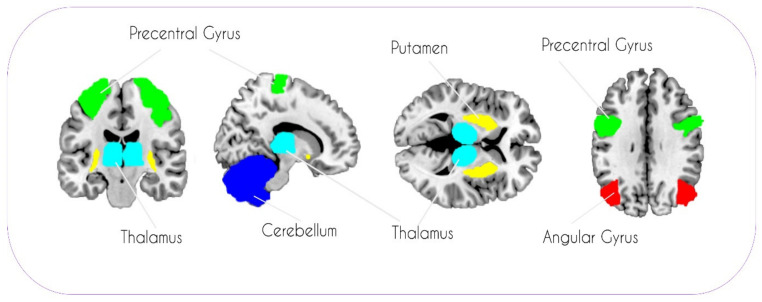
A priori selected set of regions of interest (ROIs) included in the gray-matter analysis.

For the present study, ROIs were only selected if they were reported to differ significantly between at least one trans- and one cisgender group in several of the 11 previously published studies. More precisely, the following criteria were used to ascertain our set of ROIs: (i) for each ROI, differences between trans- and cisgender participants were reported in at least three of the 11 previous studies (i.e., ROI reported in >25% of previous studies); (ii) the overall set of ROIs should span the entire brain including cortical, subcortical, and cerebellar structures; (iii) the overall set of ROIs should comprise regions from studies published by different groups, in order to reduce the influence of potential publication bias on our selection process; (iv) the total amount of ROIs selected should not be too high in order to keep the number of multiple comparisons reasonably low. Using these criteria, we selected the thalamus, putamen, cerebellum, precentral gyrus, and the angular gyrus (AG) for further analyses. Except for the thalamus, separate ROIs were used for the left and right hemispheres, resulting in nine ROIs. These a priori defined regions are visualized in Figure 1. Masks for these anatomical ROIs were obtained from the WFU Pickatlas toolbox (Wake Forest School of Medicine, Winston Salem). Mean parameter estimates of each ROI for each participant were then extracted using MarsBaR (http://marsbar.sourceforge.net/ (accessed on 23 September 2020) and transferred to SPSS 25 (SPSS Inc., Chicago, IL, USA) for further analysis.

### 2.5. Data Analysis

The primary aim of this study was to examine gender identity effects, i.e., differences between all cisgender and all transgender participants. Using multivariate analysis of variance (MANOVA), we calculated a model with two groups (cis, trans) as the between-subject factor and the nine ROIs as dependent variables. Additionally, we calculated the same model with four groups (CW, CM, TM, TW) as the between-subject factor and the nine ROIs as dependent variables. The four-group model was needed to examine the directionality of ensuing effects and to increase comparability with previous findings. In line with previous studies, both models included the total intracranial volume (TIV), as well as the age and the level of education, as covariates of no interest. Inclusion of these covariates is relevant since GMV has been shown to vary substantially depending on these factors. Furthermore, given that TIV was accounted for, all GMV differences reported here should be interpreted as being relative rather than absolute. To account for multiple comparisons when examining pairwise comparisons in the nine different ROIs, we applied Bonferroni correction. This method was chosen because it represents a rather conservative correction method necessary for our aim of pinpointing only the most consistent ROIs while keeping false-positive results to a minimum.

## 3. Results

### 3.1. Participants

An overview of demographic data for all participants can be seen in Table 1. Three separate univariate ANOVAs were used to test for group differences of demographic variables and TIV. The four groups (CM, CW, TM, TW) were used as the between-subject factor, and a significant main effect for “group” (*p* < 0.05) was found for all three ANOVAs. For age as dependent variable, we found the following group differences: CM > TM (*p* = 0.002), CW > TM (*p* = 0.009), and TW > TM (*p* = 0.001). With the level of education as a dependent variable, the following group differences were observed: CM > TM (*p* = 0.021) and CW > TM (*p* = 0.021). Regarding TIV as a dependent variable, group differences were observed for CM > CW (*p* < 0.001), CM > TM (*p* < 0.001), TW > CW (*p* < 0.001), and TW > TM (*p* < 0.001).

### 3.2. GMV Differences

**Figure 2:**
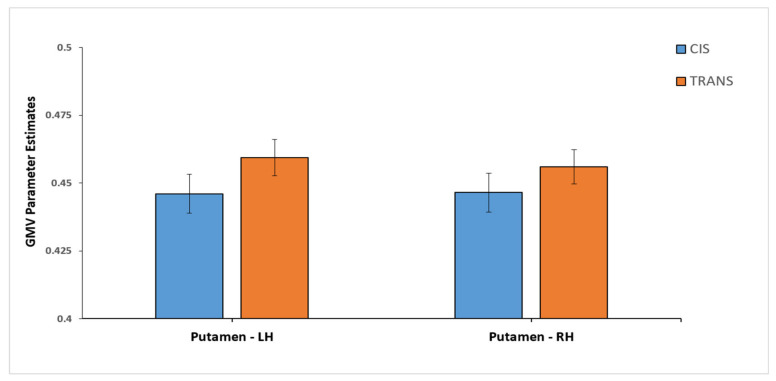
Differences in gray matter volumes (GMV) for the putamen, depicted separately for cisgender and transgender participants. The *y*-axis depicts values representing parameters estimates for GMV. The cis group includes both cismen and ciswomen and the trans group includes both transwomen and transmen. Significant GMV group differences, at *p* < 0.05 (Bonferroni corrected), were present for both the left and the right putamen. LH = left hemisphere; RH = right hemisphere.

**Figure 3:**
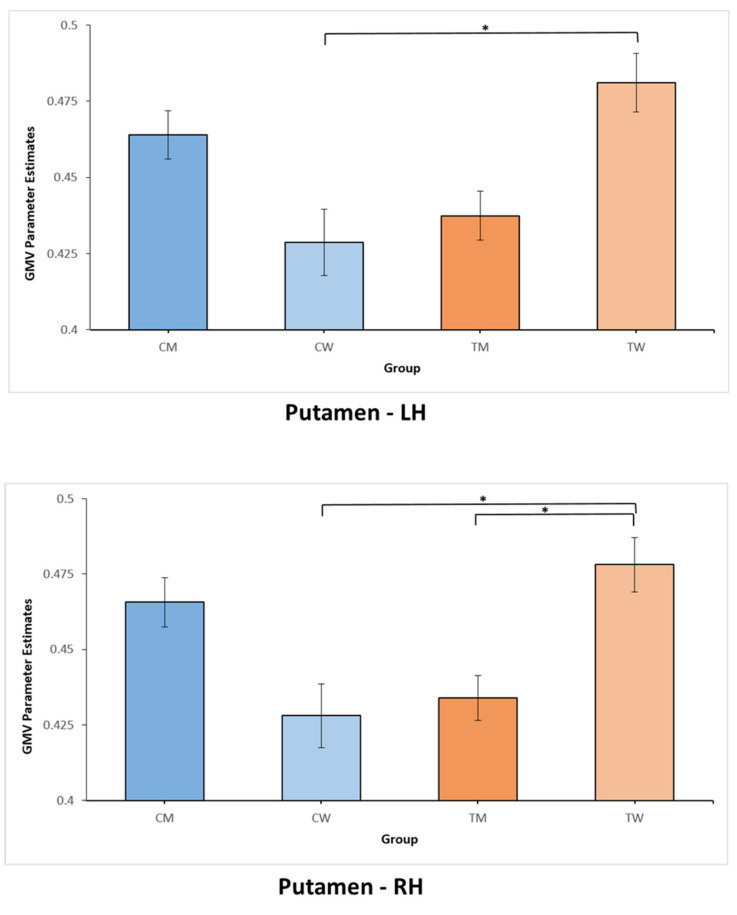
Differences in gray matter volumes (GMV) for the putamen, depicted separately for cismen, ciswomen, transmen, and transwomen. The *y*-axis depicts values representing parameters estimates for GMV. Asterisks (*) indicate significant GMV group differences, at *p* < 0.05 (Bonferroni corrected). CW = cisgender women; CM = cisgender men; LH = left hemisphere; RH = right hemisphere; TM = transmen; TW = transwomen.

A significant group effect on the multivariate level was not found, neither in the two-group MANOVA with cis and trans, nor in the four-group MANOVA with CW, CM, TM, and TW. Upon further investigation of univariate effects, the two-group MANOVA (cis vs. trans) revealed a significant group effect only for the left (F(1, 110) = 5.22, *p* = 0.024, η^2^ = 0.045) and right (F(1, 110) = 3.96, *p* = 0.049, η^2^ = 0.035) putamen. Inspecting the pairwise comparisons revealed larger GMV for trans compared to cis in both the left (mean difference = 0.018, p_Bonferroni_ = 0.024) and the right putamen (mean difference = 0.014, p_Bonferroni_ = 0.049). Figure 2 provides further visualization of these group difference in the left and right putamen. For better comparability with previous studies and in order to analyze specific differences between all groups of participants, we also examined the univariate group effects from the four-group MANOVA. Corroborating the aforementioned results from the two-group model, the four-group MANOVA also yielded a significant group effect only for the left (F(3, 108) = 4.09, *p* = 0.009, η^2^ = 0.102) and right (F(3, 108) = 4.65, *p* = 0.004, η^2^ = 0.114) putamen. When examining the pairwise comparisons for all four groups, we saw larger GMV for TW as compared to CW in both hemispheres (left putamen: mean difference = 0.035, p_Bonferroni_ = 0.014; right putamen: mean difference = 0.032, p_Bonferroni_ = 0.009). Moreover, larger GMV was found for TW relative to TM (mean difference = 0.031, p_Bonferroni_ = 0.015) in the right putamen. Group differences for the four-group model are shown in Figure 3. For better overview and full transparency of GMV results, we present all means and standard deviations for all nine ROIs in Table 2 (depicted separately for cis and trans) and Table 3 (depicted separately for CM, CW, TM, and TW).

## 4. Discussion

Optimizing therapeutic interventions and implementing individualized precision medicine approaches to predict when and how a person is affected by GI are difficult goals to achieve and seldom based on neurobiological mechanistic insights. Such approaches are currently not feasible due to a limited understanding of the underlying brain structural (and functional) mechanisms. Addressing this situation, we set out to investigate and replicate GMV differences between cisgender individuals and transgender individuals with GI. The aim of the present study was to identify brain regions which have the potential to qualify as structural biomarkers of GI in the future. More specifically, our goal was to identify one or several specific ROIs that were significantly different between cis- and transgender in multiple previous studies and the present study. Our results indicate the putamen as the only region fitting these criteria.

Distinct neuroanatomical characteristics of the putamen were associated with gender identity, both in the majority of previous studies and in the present investigation. Importantly, the observed GMV differences in the putamen are not primarily explained by a pure biological sex effect, as we did not find significant differences between CW and CM in our study. The gender identity effect in the putamen in the present findings corroborates six of the seven previous studies reporting GMV differences in this region. Except for Savic and colleagues [10], six previous studies [9,32,33,34,35,36] and the present study found larger GMV for trans- as compared to cisgender participants. It is beyond the scope of the present study to investigate the reasons for this discrepancy between Savic and colleagues [10] and the remaining studies. We suggest that confounding factors such as sexual orientation, GAHT, and genetic profiles might play a role. With respect to sexual orientation, Savic and colleagues [10] were the only ones to include strictly heterosexual TW; all transgender participants in their study were assigned male at birth, wanted to live as women, and were sexually attracted to ciswomen. Furthermore, they specifically excluded TW who had already received GAHT. Similar to previous studies, we suggest that the observed putamen effect is most likely not influenced by GAHT status. In different studies, increased GMV in this region was found both for TW who had not started GAHT [9] and for TW who were already receiving GAHT (including the present study) [34]. Another aspect that further complicates the issue of GAHT influences in neuroanatomical studies of GI was reported by Kranz and colleagues [37]. In their review, the authors pointed out that the different hormones given to TM and TW might have opposite effects on GMV. Testosterone treatment in TM likely has a primarily anticatabolic effect on brain volume, resulting in increased GMV in subcortical regions such as the thalamus and the pallidum. On the other hand, estrogen and antiandrogen treatment in TW seems to reduce GMV in the hypothalamus, thalamus, pallidum, and hippocampus.

Our ability to replicate previous findings for the putamen seems to corroborate the involvement of this brain region in GI. However, before we can determine with certainty that the putamen is the exact anatomical region which most consistently differs between trans- and cisgender participants, further confirmation and extension of our results is needed. Importantly, researchers should strive to replicate these findings in large-scale datasets (e.g., UK Biobank). The rather small sample sizes of the present and previous studies preclude the detection of very subtle, but important, neuroanatomical differences. Moreover, additional methodologies are needed to support such a claim, including multimodal approaches and combinations of data from multiple studies or sites in a meta-analytic framework. Whether such structural changes, as observed in the present study, are innate or acquired in transgender participants with GI remains to be clarified by neurodevelopmental and longitudinal studies. Most importantly, our results clearly align with the general pattern and direction of previous findings: larger GMV in putamen for trans as compared to cis in general and larger GMV in the putamen for TW as compared to CW and TM. More specifically, our results and the majority of previous findings illustrate that the observed putamen effects are strongly influenced by larger GMV of the TW group as compared to all other groups. It seems that the specificity, usefulness, and clinical relevance of the putamen as a neuroanatomical marker might be highest for TW relative to TM or transgender individuals in general. Future studies must further evaluate the discriminatory power of these effects both in TW with and without GAHT.

However, what makes the putamen especially relevant for transgender and GI? In accordance with previous studies [10,33], we suggest that the specific role for the putamen in GI is most likely related to the general function of this brain region in processing body representations and the own body image. Such an explanation seems reasonable, considering that the strong feeling of being trapped in the wrong body and persistent discomfort with one’s physical appearance represent key features of GI. The putamen is one of the brain regions which is crucial for the integration of somatosensory and motoric information relevant to coordinate context-dependent motor and cognitive responses [38]. Previous functional MRI studies revealed that the putamen generates responses to painful stimuli and processes inputs from different body parts via somatotopically organized response mappings [39,40,41]. Thus, fMRI studies have confirmed the somatotopic arrangement of both motor responses and nociceptive information in the putamen [42,43]. Together with the cerebellum, somatosensory and motor cortices, the thalamus, and the basal ganglia, the putamen is thought to be directly involved in the formation of the own body image [44]. Accordingly, several fMRI studies observed activation patterns related to body image dissatisfaction in the putamen in anorexia nervosa [45,46]. Furthermore, the putamen, in combination with other multisensory integration areas, enables us to experience our body as distinct from the rest of the outside world, thus creating a unitary experience of whole-body ownership [47]. In this context, we can only speculate as to why the putamen effects are most strongly found in the TW group. Putamen volumes might be specifically linked to the level of body dissatisfaction in TW because these individuals are convinced that a corresponding, female body image represents an essential part of being a woman and that a female physical appearance is of importance for cis- and transwomen. It was previously demonstrated that the constant evaluation of the own body image may be of much stronger significance in transgender individuals, especially TW, because of a strong focus on low social acceptance of sexually dimorphic face and body features in these individuals [48].

From childhood on, our body image is reinforced via multisensory integration of external and internal stimuli which in most cases leads to a congruence between self-perception and own body image. The dissociative feelings experienced by transgender individuals with GI could, thus, be due to neurodevelopmental changes in the putamen and other brain regions processing own body perception. The formation of a congruent body image represents a very delicate and complex developmental milestone, involving neuronal processes enabling somato-perception, somato-representation, and alignment between the physical and the psychological self. If neural networks and structures crucially involved in this process are altered in GI, this might in part also explain the discomfort between self-perception and own body image. When it comes to genetic or hormonal mechanisms causing such neurodevelopmental differences in the first place, several studies [21,49] suggested that specific α and β gene polymorphisms of sex hormone receptors are linked to GI. Given its crucial role in human brain differentiation [50], the β-estrogen receptor surely plays a key role in the development of gender identity.

In contrast to previous studies [10,15,35], we did not find GMV differences in the AG. Notably, a recent fMRI study [51] observed increased AG activity for TW as compared to CM in a gender face perception task. Moreover, they found that AG activity was significantly correlated with higher levels of body dissatisfaction. In this task-based fMRI study, the AG might be specifically activated due to its involvement in perceiving and remembering faces. The AG is involved in processing both the physical and the social aspects of faces [52,53], constituting a key region in conceptual processing [54]. Trans- and cisgender individuals might exhibit different AG activation patterns in fMRI studies with facial stimuli because evaluating faces has an inherently stronger significance in transgender individuals [48]. In our task-free, MRI data, specific processing of facial stimuli was absent. Thus, in addition to the fundamental differences in the physical parameters being assessed (blood oxygenation level dependent response vs. gray matter volumes), these methodological differences in the study design might provide a potential explanation for the discrepancy between the present MRI and previous fMRI results for the AG.

Our findings should be considered in light of several limitations that future studies could address and expand upon. While the own body perception hypothesis might provide a fitting theoretical framework to interpret our results, other explanations and interpretations are possible as well. We do not intend to present our results as a definite proof or unequivocal support of the own body perception hypothesis. Specifically, the observed difference in putamen GMV between TW and TM is difficult to explain if differences in own body perception are assumed for all transgender individuals. Furthermore, we cannot discern the specific influence of GAHT on our results as we included both individuals with and without concurrent GAHT. While theoretically possible, we did not split the transgender groups further as this would have resulted in smaller group sizes insufficiently powered to robustly detect statistical effects. As the goal of the present study was to investigate brain structural differences between cis- and transgender individuals, we did not systematically assess hormonal levels for the present sample. We acknowledge, however, that GAHT might represent an important factor influencing neuroimaging correlates of GI. Large-scale multicenter studies are currently under way, investigating how neuroimaging parameters, such as GMV, resting-state functional connectivity, task-based fMRI, and anatomical connectivity, change in the course of GAHT. We believe that only such longitudinal designs, with repeated MR measurements before and after the start of GAHT, are suitable to analyze and quantify specific GAHT effects. Furthermore, sexual orientation of cis- and transgender participants has to be controlled for and assessed in a systematic manner in future studies. While it seems that some preliminary findings point to the existence of brain anatomical differences due to sexual orientation [55,56,57,58], others found no difference [33]. Examining these five previous studies with respect to sexual orientation effects in the putamen, two found such effects and three did not. Thus, one cannot exclude the possibility that findings in the putamen in the present study are also influenced by sexual orientation to some degree. Thus, sexual orientation should be accounted for in all future neuroimaging transgender studies, with the aim of delineating and separating the effects of gender identity and sexual orientation more precisely. Scientific journals and funding agencies might support this process by explicitly asking researchers to include the sexual orientation of all individuals in studies on sex, sexual orientation, and gender identity.

Our results have some crucial implications for transgender and GI. Issues related to self-identity and medical interventions in individuals with GI are important medical and societal challenges for the 21st century, specifically considering the high suicide risk associated with GI [16,59,60]. As GI can have a rather early onset in many patients, it becomes important to examine the neurodevelopmental trajectory of these GMV differences in the putamen in youth and adolescence. Further changes in the putamen over the course of GAHT have to be delineated in longitudinal studies. Lastly, it should be noted that tangible efforts to design predictive modelling approaches in GI are already underway with promising results from others [32,61] and our own group [22]. In the future, these parallel efforts have to be combined and tested in larger samples in order to create robust multimodal modeling frameworks incorporating GMV, cortical thickness, brain activation, and resting-state functional connectivity profiles. For many transgender individuals with GI, GAHT is followed by gender-affirming surgery [62]. Whereas some patients will substantially benefit from GAHT and gender-affirming surgery, others exhibit a very poor treatment response. Therefore, predictive tools supporting medical professionals in their decision whether or not to apply GAHT and gender-affirming surgery are highly warranted. Personalizing treatment in GI would also mean to be able to identify individuals for whom it may be optimal to prescribe additional treatments and specifically tailor application of testosterone, hormonal antagonists, or alternate types of sex hormones. Without substantial advances in these areas, individualized and precision medicine approaches cannot be implemented, and an increasing number of patients will continue to suffer from health issues associated with GI. Thus, the present study provides a potential replication of previous results while also exemplifying the need for clinicians and scientists to further increase their understanding of the specific underlying neurobiological mechanisms of GI.

## Figures and Tables

**Table 1:** Demographic data for cisgender and transgender participants.

	CW (*n* = 25)	CM (*n* = 24)	TM (*n* = 33)	TW (*n* = 33)
Age	31 (11)	33 (11)	24 (7)	33 (13)
Years of education	15 (3)	15 (3)	14 (3)	14 (3)
TIV	1460 (115)	1605 (97)	1412 (107)	1567 (102)

The table gives relevant demographic information (means, with standard deviations in brackets) about participants, divided by groups. CW = cisgender women; CM = cisgender men; TIV = total intracranial volume; TM = transmen; TW = transwomen.

**Table 2:** Gray matter volumes (GMVs) of all ROIs, depicted separately for cisgender and transgender participants.

	CIS (*n* = 49)	TRANS (*n* = 66)
Putamen (L) *	0.446 (0.05)	0.4593 (0.055)
Putamen (R) *	0.4465 (0.05)	0.4560 (0.051)
Precentral Gyrus (L)	0.3341 (0.041)	0.3338 (0.04)
Precentral Gyrus (R)	0.3232 (0.038)	0.3180 (0.038)
Thalamus	0.3699 (0.045)	0.3677 (0.042)
AG (L)	0.3904 (0.058)	0.3821 (0.059)
AG (R)	0.3491 (0.052)	0.3425 (0.045)
Cerebellum (L)	0.4048 (0.037)	0.4023 (0.033)
Cerebellum (R)	0.4105 (0.039)	0.4086 (0.035)

The table presents mean values per group for GMV (with standard deviations in brackets) for cis- and transgender participants. All values represent parameters estimates for GMV. The cis group includes both cismen and ciswomen and the trans group includes both transwomen and transmen. Asterisks (*) indicate significant GMV group differences for this region, trans > cis, at *p* < 0.05 (Bonferroni corrected).

**Table 3:** Gray matter volumes for all ROIs, depicted separately for cis men, cis women, transmen, and transwomen.

	CW (*n* = 25)	CM (*n* = 24)	TM (*n* = 33)	TW (*n* = 33)
Putamen (L) ^(a)^	0.4287 (0.055)	0.464 (0.039)	0.4374 (0.046)	0.4812 (0.054)
Putamen (R) ^(a)&(b)^	0.4280 (0.053)	0.4657 (0.04)	0.4339 (0.042)	0.4781 (0.051)
Precentral Gyrus (L)	0.3176 (0.036)	0.3513 (0.04)	0.3305 (0.029)	0.3371 (0.049)
Precentral Gyrus (R)	0.31 (0.039)	0.3369 (0.033)	0.317 (0.034)	0.3189 (0.041)
Thalamus	0.3622 (0.038)	0.378 (0.05)	0.3679 (0.042)	0.3675 (0.042)
AG (L)	0.3727 (0.05)	0.4088 (0.062)	0.3661 (0.05)	0.3982 (0.064)
AG (R)	0.3263 (0.045)	0.373 (0.05)	0.3353 (0.045)	0.3497 (0.045)
Cerebellum (L)	0.3945 (0.036)	0.4155 (0.036)	0.3964 (0.034)	0.4081 (0.032)
Cerebellum (R)	0.3987 (0.036)	0.4227 (0.038)	0.4021 (0.036)	0.4152 (0.034)

The table presents mean values per group for GMV (with standard deviations in brackets) for cis men, cis women, transmen and transwomen. All values represent parameters estimates for GMV. CW = cisgender women; CM = cisgender men; TM = transmen; TW = transwomen. (**a**) TW > CW, significant group differences at *p* < 0.05 (Bonferroni corrected). (**b**) TW > TM, significant group differences, at *p* < 0.05 (Bonferroni corrected).

## Data Availability

The data presented in this study are available on request from the corresponding author. The data are not publicly available due to policies on data protection and privacy enforced by the RWTH Aachen University.

## References

[1] American Psychiatric Association (2013). Diagnostic and Statistical Manual of Mental Disorders.

[2] Heylens G., Elaut E., Kreukels B.P.C., Paap M.C.S., Cerwenka S., Richter-Appelt H., Cohen-Kettenis P.T., Haraldsen I.R., De Cuypere G. (2014). Psychiatric characteristics in transsexual individuals: Multicentre study in four European countries. Br. J. Psychiatry.

[3] Safer J.D., Tangpricha V. (2019). Care of the Transgender Patient. Ann. Intern. Med..

[4] Valentine S.E., Shipherd J.C. (2018). A systematic review of social stress and mental health among transgender and gender non-conforming people in the United States. Clin. Psychol. Rev..

[5] Reed G.M., Drescher J., Krueger R.B., Atalla E., Cochran S.D., First M.B., Cohen-Kettenis P.T., Arango-de Montis I., Parish S.J., Cottler S. (2016). Disorders related to sexuality and gender identity in the ICD-11: Revising the ICD-10 classification based on current scientific evidence, best clinical practices, and human rights considerations. World Psychiatry.

[6] Van Caenegem E., Wierckx K., Elaut E., Buysse A., Dewaele A., Van Nieuwerburgh F., De Cuypere G., T’Sjoen G. (2015). Prevalence of Gender Nonconformity in Flanders, Belgium. Arch. Sex. Behav..

[7] Kuyper L., Wijsen C. (2014). Gender Identities and Gender Dysphoria in the Netherlands. Arch. Sex. Behav..

[8] Arcelus J., Bouman W.P., Van Den Noortgate W., Claes L., Witcomb G., Fernandez-Aranda F. (2015). Systematic Review and Meta-Analysis of Prevalence Studies in Transsexualism. Eur. Psychiatry.

[9] Luders E., Sánchez F.J., Gaser C., Toga A.W., Narr K.L., Hamilton L.S., Vilain E. (2009). Regional gray matter variation in male-to-female transsexualism. Neuroimage.

[10] Savic I., Arver S. (2011). Sex Dimorphism of the Brain in Male-to-Female Transsexuals. Cereb. Cortex.

[11] Spizzirri G., Duran F.L.S., Chaim-Avancini T.M., Serpa M.H., Cavallet M., Pereira C.M.A., Santos P.P., Squarzoni P., da Costa N.A., Busatto G.F. (2018). Grey and white matter volumes either in treatment-naïve or hormone-treated transgender women: A voxel-based morphometry study. Sci. Rep..

[12] Zhou J.-N., Hofman M.A., Gooren L.J.G., Swaab D.F. (1995). A sex difference in the human brain and its relation to transsexuality. Nature.

[13] Garcia-Falgueras A., Swaab D.F. (2008). A sex difference in the hypothalamic uncinate nucleus: Relationship to gender identity. Brain.

[14] Swaab D. (2004). Sexual differentiation of the human brain: Relevance for gender identity, transsexualism and sexual orientation. Gynecol. Endocrinol..

[15] Simon L., Kozák L.R., Simon V., Czobor P., Unoka Z., Szabó Á., Csukly G. (2013). Regional Grey Matter Structure Differences between Transsexuals and Healthy Controls—A Voxel Based Morphometry Study. PLoS ONE.

[16] Mueller S.C., De Cuypere G., T’Sjoen G. (2017). Transgender research in the 21st century: A selective critical review from a neurocognitive perspective. Am. J. Psychiatry.

[17] Hoekzema E., Schagen S.E.E., Kreukels B.P.C., Veltman D.J., Cohen-Kettenis P.T., Delemarre-van de Waal H., Bakker J. (2015). Regional volumes and spatial volumetric distribution of gray matter in the gender dysphoric brain. Psychoneuroendocrinology.

[18] Manzouri A., Kosidou K., Savic I. (2017). Anatomical and Functional Findings in Female-to-Male Transsexuals: Testing a New Hypothesis. Cereb. Cortex.

[19] Burke S.M., Manzouri A.H., Savic I. (2017). Structural connections in the brain in relation to gender identity and sexual orientation. Sci. Rep..

[20] Wallien M.S.C., Zucker K.J., Steensma T.D., Cohen-Kettenis P.T. (2008). 2D:4D finger-length ratios in children and adults with gender identity disorder. Horm. Behav..

[21] Guillamon A., Junque C., Gómez-Gil E. (2016). A Review of the Status of Brain Structure Research in Transsexualism. Arch. Sex. Behav..

[22] Clemens B., Derntl B., Smith E., Junger J., Neulen J., Mingoia G., Schneider F., Abel T., Bzdok D., Habel U. (2020). Predictive Pattern Classification Can Distinguish Gender Identity Subtypes from Behavior and Brain Imaging. Cereb. Cortex.

[23] Joel D., Berman Z., Tavor I., Wexler N., Gaber O., Stein Y., Shefi N., Pool J., Urchs S., Margulies D.S. (2015). Sex beyond the genitalia: The human brain mosaic. Proc. Natl. Acad. Sci. USA.

[24] Clemens B., Junger J., Pauly K., Neulen J., Neuschaefer-Rube C., Frölich D., Mingoia G., Derntl B., Habel U. (2017). Male-to-female gender dysphoria: Gender-specific differences in resting-state networks. Brain Behav..

[25] Junger J., Pauly K., Bröhr S., Birkholz P., Neuschaefer-Rube C., Kohler C., Schneider F., Derntl B., Habel U. (2013). Sex matters: Neural correlates of voice gender perception. Neuroimage.

[26] Smith E., Junger J., Pauly K., Kellermann T., Neulen J., Neuschaefer-Rube C., Derntl B., Habel U. (2018). Gender incongruence and the brain—Behavioral and neural correlates of voice gender perception in transgender people. Horm. Behav..

[27] Wittchen H.U., Zaudig M., Fydrich T. (1997). Strukturiertes Klinisches Interview für DSM-IV (SKID-I und SKID-II).

[28] Mietchen D. (2009). Computational morphometry for detecting changes in brain structure due to development, aging, learning, disease and evolution. Front. Neuroinform..

[29] Honea R., Crow T.J., Passingham D., Mackay C.E. (2005). Regional Deficits in Brain Volume in Schizophrenia: A Meta-Analysis of Voxel-Based Morphometry Studies. Am. J. Psychiatry.

[30] White Hughto J.M., Reisner S.L., Pachankis J.E. (2015). Transgender stigma and health: A critical review of stigma determinants, mechanisms, and interventions. Soc. Sci. Med..

[31] Pol H.E.H., Cohen-Kettenis P.T., Van Haren N.E.M., Peper J.S., Brans R.G.H., Cahn W., Schnack H.G., Gooren L.J.G., Kahn R.S. (2006). Changing your sex changes your brain: Influences of testosterone and estrogen on adult human brain structure. Eur. J. Endocrinol..

[32] Flint C., Förster K., Koser S.A., Konrad C., Zwitserlood P., Berger K., Hermesdorf M., Kircher T., Nenadic I., Krug A. (2020). Biological sex classification with structural MRI data shows increased misclassification in transgender women. Neuropsychopharmacology.

[33] Manzouri A., Savic I. (2019). Possible Neurobiological Underpinnings of Homosexuality and Gender Dysphoria. Cereb. Cortex.

[34] Mueller S.C., Landré L., Wierckx K., T’Sjoen G. (2017). A Structural Magnetic Resonance Imaging Study in Transgender Persons on Cross-Sex Hormone Therapy. Neuroendocrinology.

[35] Sorouri Khorashad B., Khazai B., Talaei A., Acar F., Hudson A.R., Borji N., Saberi H., Aminzadeh B., Mueller S.C. (2020). Neuroanatomy of transgender persons in a Non-Western population and improving reliability in clinical neuroimaging. J. Neurosci. Res..

[36] Zubiaurre-Elorza L., Junque C., Gomez-Gil E., Segovia S., Carrillo B., Rametti G., Guillamon A. (2013). Cortical Thickness in Untreated Transsexuals. Cereb. Cortex.

[37] Kranz G.S., Zhang B.B.B., Handschuh P., Ritter V., Lanzenberger R. (2020). Gender-affirming hormone treatment—A unique approach to study the effects of sex hormones on brain structure and function. Cortex.

[38] Bingel U., Quante M., Knab R., Bromm B., Weiller C., Büchel C. (2002). Subcortical structures involved in pain processing: Evidence from single-trial fMRI. Pain.

[39] Derbyshire S.W.G., Whalley M.G., Stenger V.A., Oakley D.A. (2004). Cerebral activation during hypnotically induced and imagined pain. Neuroimage.

[40] Hui K.K., Liu J., Chen A.J.W., Makris N., Kennedy D.N., Moore C., Gollub R.L., Rosen B.R., Kwong K.K. (1998). Acupuncture Modulates the Limbic System and Subcortical Gray Structures of the Human Brain—Direct Evidence by fMRI. Neuroimage.

[41] Coghill R.C., Gilron I., Iadarola M.J. (2001). Hemispheric lateralization of somatosensory processing. J. Neurophysiol..

[42] Bingel U., Gläscher J., Weiller C., Büchel C. (2004). Somatotopic Representation of Nociceptive Information in the Putamen: An Event-related fMRI Study. Cereb. Cortex.

[43] Maillard L., Ishii K., Bushara K., Waldvogel D., Schulman A.E., Hallett M. (2000). Mapping the basal ganglia: fMRI evidence for somatotopic representation of face, hand, and foot. Neurology.

[44] Fontan A., Cignetti F., Nazarian B., Anton J.-L., Vaugoyeau M., Assaiante C. (2017). How does the body representation system develop in the human brain?. Dev. Cogn. Neurosci..

[45] Vocks S., Schulte D., Busch M., Grönemeyer D., Herpertz S., Suchan B. (2011). Changes in neuronal correlates of body image processing by means of cognitive-behavioural body image therapy for eating disorders: A randomized controlled fMRI study. Psychol. Med..

[46] Mohr H.M., Zimmermann J., Röder C., Lenz C., Overbeck G., Grabhorn R. (2010). Separating two components of body image in anorexia nervosa using fMRI. Psychol. Med..

[47] Petkova V.I., Björnsdotter M., Gentile G., Jonsson T., Li T.Q., Ehrsson H.H. (2011). From part-to whole-body ownership in the multisensory brain. Curr. Biol..

[48] Fisher A.D., Castellini G., Ristori J., Casale H., Cassioli E., Sensi C., Fanni E., Amato A.M.L., Bettini E., Mosconi M. (2016). Cross-Sex Hormone Treatment and Psychobiological Changes in Transsexual Persons: Two-Year Follow-Up Data. J. Clin. Endocrinol. Metab..

[49] Raznahan A., Lee Y., Stidd R., Long R., Greenstein D., Clasen L., Addington A., Gogtay N., Rapoport J.L., Giedd J.N. (2010). Longitudinally mapping the influence of sex and androgen signaling on the dynamics of human cortical maturation in adolescence. Proc. Natl. Acad. Sci. USA.

[50] Fernández R., Guillamon A., Cortés-Cortés J., Gómez-Gil E., Jácome A., Esteva I., Almaraz M.C., Mora M., Aranda G., Pásaro E. (2018). Molecular basis of Gender Dysphoria: Androgen and estrogen receptor interaction. Psychoneuroendocrinology.

[51] Fisher A.D., Ristori J., Castellini G., Cocchetti C., Cassioli E., Orsolini S., Sensi C., Romani A., Mazzoli F., Cipriani A. (2020). Neural Correlates of Gender Face Perception in Transgender People. J. Clin. Med..

[52] Hölig C., Föcker J., Best A., Röder B., Büchel C. (2017). Activation in the angular gyrus and in the pSTS is modulated by face primes during voice recognition. Hum. Brain Mapp..

[53] Allison T., Puce A., McCarthy G. (2000). Social perception from visual cues: Role of the STS region. Trends Cogn. Sci..

[54] Binder J.R., Desai R.H. (2011). The neurobiology of semantic memory. Trends Cogn. Sci..

[55] Abé C., Johansson E., Allzén E., Savic I. (2014). Sexual Orientation Related Differences in Cortical Thickness in Male Individuals. PLoS ONE.

[56] Ponseti J., Siebner H.R., Klöppel S., Wolff S., Granert O., Jansen O., Mehdorn H.M., Bosinski H.A. (2007). Homosexual Women Have Less Grey Matter in Perirhinal Cortex than Heterosexual Women. PLoS ONE.

[57] Witelson S.F., Kigar D.L., Scamvougeras A., Kideckel D.M., Buck B., Stanchev P.L., Bronskill M., Black S. (2008). Corpus Callosum Anatomy in Right-Handed Homosexual and Heterosexual Men. Arch. Sex. Behav..

[58] Votinov M., Goerlich K.S., Puiu A.A., Smith E., Nickl-Jockschat T., Derntl B., Habel U. (2021). Brain structure changes associated with sexual orientation. Sci. Rep..

[59] Maguen S., Shipherd J.C. (2010). Suicide risk among transgender individuals. Psychol. Sex..

[60] Narang P., Sarai S.K., Aldrin S., Lippmann S. (2018). Suicide Among Transgender and Gender-Nonconforming People. Prim. Care Companion CNS Disord..

[61] Baldinger-Melich P., Urquijo Castro M.F., Seiger R., Ruef A., Dwyer D.B., Kranz G.S., Klöbl M., Kambeitz J., Kaufmann U., Windischberger C. (2020). Sex Matters: A Multivariate Pattern Analysis of Sex- and Gender-Related Neuroanatomical Differences in Cis- and Transgender Individuals Using Structural Magnetic Resonance Imaging. Cereb. Cortex.

[62] Van de Grift T.C., Elaut E., Cerwenka S.C., Cohen-Kettenis P.T., Kreukels B.P.C. (2018). Surgical Satisfaction, Quality of Life, and Their Association After Gender-Affirming Surgery: A Follow-up Study. J. Sex Marital Ther..

